# Electrochemical promotion of propane oxidation on Pt deposited on a dense β″-Al_2_O_3_ ceramic Ag^+^ conductor

**DOI:** 10.3389/fchem.2013.00013

**Published:** 2013-08-30

**Authors:** Mihalis N. Tsampas, Anastasios Kambolis, Emil Obeid, Leonardo Lizarraga, Foteini M. Sapountzi, Philippe Vernoux

**Affiliations:** Centre National de la Recherche Scientifique, Institut de Recherches sur la Catalyse et l'Environnement de Lyon, UMR 5256, Université de Lyon, Université Claude Bernard Lyon 1Villeurbanne, France

**Keywords:** EPOC, NEMCA effect, propane combustion, β″-Al_2_O_3_ (Ag^+^) electrochemical catalysts, Ag^+^ cations

## Abstract

A new kind of electrochemical catalyst based on a Pt porous catalyst film deposited on a β″-Al_2_O_3_ ceramic Ag^+^ conductor was developed and evaluated during propane oxidation. It was observed that, upon anodic polarization, the rate of propane combustion was significantly electropromoted up to 400%. Moreover, for the first time, exponential increase of the catalytic rate was evidenced during galvanostatic transient experiment in excellent agreement with EPOC equation.

## Introduction

Ionic conductors (i.e., materials that can selectively transport ions, also called electrolytes) are widely used in batteries, fuel cells, sensors and in gas separation technologies. Solid electrolytes with conducting ions of O^2−^, H^+^, Li^+^, K^+^, Na^+^, Ag^+^, F^−^, Cu^+^, or Cl^−^ have been reported in the last decades (Hamann et al., 1998; Vayenas et al., 2001; Vielstich et al., 2003; Vernoux et al., 2013). The importance of these materials as catalytic carriers in heterogeneous catalysis has been obvious since the demonstration by Vayenas and coworkers (Vayenas et al., 2001; Vayenas, 2011; Vernoux et al., 2013) that the conducting ionic species can significantly promote the catalytic activity of metal or metal oxide nanoparticles, via the non-Faradaic Electrochemical Modification of Catalytic Activity (NEMCA) effect, also called Electrochemical Promotion of Catalysis (EPOC).

In EPOC studies, the catalyst active phase (conductive catalyst-electrode) is in contact with an ionic conductor-support (in an electrochemical cell configuration). Application of electrical currents or imposition of potentials between the catalyst film and a counter electrode (catalytically inactive) can cause significant alterations in catalytic properties (Vayenas et al., 2001; Vayenas, 2011; Vernoux et al., 2013).

A key parameter in EPOC studies is the catalyst potential *U*_*WR*_. The subscript “*WR*” denotes the potential of the working (“*W*”) electrode (which also serves as the catalyst) with respect to a reference “*R*” electrode. Experimental (i.e., *in situ* work function measurements) (Vayenas et al., 1990, 2001; Tsiplakides and Vayenas, 2001) and theoretical studies (Tsiplakides and Vayenas, 2001; Riess and Vayenas, 2003) have shown that there exists (over wide temperature ranges) an one-to-one correlation between the change in *U*_*WR*_ and the concomitant change in the work function, Φ, of the gas exposed, i.e., catalytically active, catalyst surface:

(1) $$\Delta \Phi =e\Delta U_{WR}$$

Thus, in electrochemical promotion studies, upon varying the catalyst potential, *U*_*WR*_, the catalyst work function, Φ, is also modified. Increasing coverage of electron acceptor (electronegative) promoting species increases the catalyst potential and work function while increasing coverage of electron donor (electropositive) promoting species decreases both the catalyst potential and the work function (Stoukides and Vayenas, 1981; Vayenas et al., 2001).

Since the discovery of EPOC in the early 80 s (Stoukides and Vayenas, 1981), more than 100 different catalytic systems (oxidations, hydrogenations, dehydrogenations, isomerizations, decompositions) have been electrochemically promoted on Pt, Pd, Rh, Ag, Au, Ni, IrO_2_, RuO_2_ catalysts deposited on O^2−^ (YSZ), Na^+^ (β″-Al_2_O_3_, NaSiCon), K^+^ (β″-Al_2_O_3_), H^+^ (CaZr_0.9_In_0.1_O_3−α_, Nafion), F^−^ (CaF_2_), aqueous, molten salt and mixed ionic-electronic (TiO_2_, CeO_2_, La_0.6_Sr_0.4_Co_0.2_Fe_0.8_O_3−δ_/Ce_0.9_Gd_0.1_O_1.95_ composite) conductors (Vayenas et al., 2001; Katsaounis, 2010; Kambolis et al., 2012; Vernoux et al., 2013). EPOC does not seem to be limited to any specific type of catalytic reaction, metal catalyst or solid electrolyte, therefore, the investigation of new materials regarding the electrolyte, the promoting agent and the active catalyst phase, is very challenging. Moreover, the knowledge obtained from EPOC studies can be transferred in dispersed catalysts in terms of choosing the support for a specific reaction by choosing the appropriate ionic agent (Vernoux et al., 2009, 2013).

Silver catalysts have found excellent application to ethylene epoxidation (Dadyburjor et al., 1979; Ayame et al., 2003) and hold great promise for the SCR of NOx with hydrocarbons (Burch et al., 2002; Shimizu and Satsuma, 2006). However, sintering is a serious issue for silver catalysts (Presland et al., 1972; Plummer et al., 1987; Ruckenstein and Lee, 1988), and a complex array of factors have been linked to sintering, including the reaction gas environment (Ruckenstein and Lee, 1988), support effects (Bird et al., 2000), and surface binding and mobility (Meyer et al., 2007).

This work presents, for the first time, attempts to develop an electrochemical catalyst which allows transportation of Ag^+^ between the solid electrolyte and the Pt film. This provides a controlled, reversible, and reproducible way of altering the surface composition. The idea of this study is to utilize the phenomenon of EPOC to electrochemically control the transportation of Ag^+^ cations between the solid electrolyte and the catalyst film. Propane combustion was used as model reaction. This reaction is well-studied in the field of EPOC on Pt electrochemical catalysts deposited on O^2−^ and Na^+^ conductors. Therefore, a Pt catalyst film was deposited on β″-Al_2_O_3_ (Ag^+^). Moreover, the use of Pt film instead of Ag allows the discrimination of species coming from the support.

## Experimental

**Figure 1:**
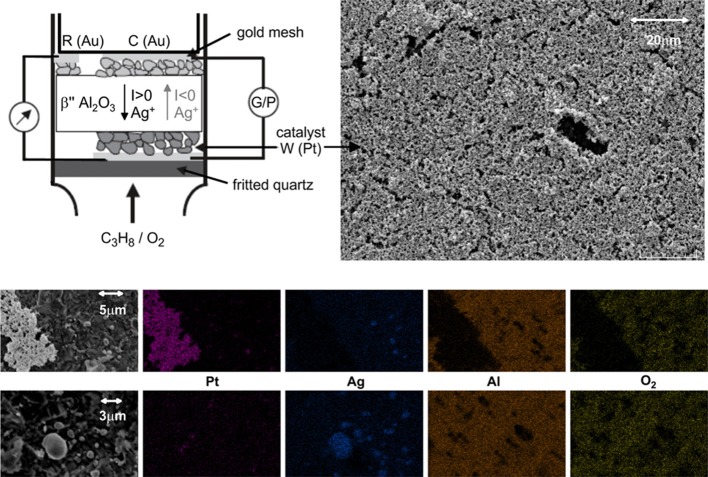
**Schematic drawing of the electrochemical catalyst Pt/β″-Al_2_O_3_ (Ag^+^) placed in the quartz reactor (the galvanostat/potentiostat is denoted as G/P) and SEM micrograph of the catalyst porous Pt film, and X-ray mapping of Pt, Ag, Al, and oxygen at two different areas of the electrode-catalyst surface**.

The electrochemical catalyst (Figure 1) consisted of a β″-Al_2_O_3_ pellet (Ionotec Ltd) Ag^+^ conductor, covered on both sides by three electrodes; the working-catalyst, the counter and the reference electrode. The Pt working electrode (0.8 cm^2^ geometric surface area and 1.9 mg loading) was deposited by application of metalorganic platinum paste (Engelhard-Clal 6926) and catalytically inactive gold counter (symmetrical to working). Reference and counter electrodes were deposited on the other side of the pellet by application of metalorganic paste, followed by calcination in air for 2 h at 450°C. The reference electrode was deposited close enough (about 3 mm) to the counter-electrode avoiding any electrical perturbation. A galvanostat/potentiostat Voltalab PGP 201 (Radiometer) was used in order to apply and measure both potential and current in the electrochemical catalyst. Voltage and current were applied and measured according to the procedure generally used in conventional three-electrode electrochemical cells. The catalyst potential, *U*_*WR*_, was measured between the working electrode and the reference electrode (Au) which may be considered as a pseudoreference since its potential does not vary significantly with the composition of the gaseous mixture, as experimentally checked.

The presence of silver in the interface of the Pt porous catalytic film and the β″-Al_2_O_3_ support was confirmed by SEM-EDX analysis carried out over the sample after catalyst performances measurements. Before the SEM-EDX analysis, the sample was cooled down under the reaction conditions from 400°C to room temperature. During this period, the sample was under positive polarization of *U*_*WR*_ = +2V. Figure 1 shows a SEM micrograph of the Pt film and a zoom in areas where the Pt film is open to the support. Figure 1 also includes a mapping of two regions for Pt, Ag, Al, and O_2_. From the mapping of elements, one can conclude that silver particles cover the area between the Pt and the β″-Al_2_O_3_. The presence of silver species in this interface can be attributed to surface diffusion during annealing the catalyst which is enhanced by the presence of oxygen (Presland et al., 1972; Baker and Skiba, 1977).

The catalytic performances of the electrochemical catalyst was evaluated in a quartz reactor, which was designed to facilitate the connection between the electrodes and the galvanostat/potentiostat, allowing the reactive mixture to reach the electrocatalyst surface. The temperature was measured with a K-type thermocouple placed in proximity to the working electrode surface. The reaction gases were mixtures of C_3_H_8_ (Air Liquide, 8000 ± 80 ppm), O_2_ (Air Liquide, 99.95%) and He (Air Liquide, 99.999%) as the vector gas. The gas composition was controlled by mass flow controllers (Brooks), with accuracy of 1%. The reaction products were analyzed by an on-line micro gas-chromatograph (R3000 SRA Instruments) and a CO_2_ IR analyzer (Horiba VA 3000). The C_3_H_8_ partial pressure in the feed was held constant during all the experiments at 0.24 kPa. The O_2_ partial pressure in the feed was kept at 1.2 kPa with total gas flow rate of 12 L/h. Carbon monoxide was never detected, according to our 10 ppm lower detection limit.

In order to quantify the magnitude of EPOC, three parameters are commonly used. First, the rate enhancement ratio, ρ:

(2) $$\rho =r/r_{o}$$

second the apparent Faradaic efficiency, Λ:

(3) $$\Lambda =\left(r-r_{o}\right)/\left(I/nF\right)$$

and third the promotional index, *PI*_Ag_:

(4) $$PI_{\text{Ag}}=(\rho -1)/\Delta \theta_{\text{Ag}}$$

where *r* is the electropromoted catalytic rate, *r*_o_ the open-circuit catalytic rate, *I* the applied current, and *n* the number of exchanged electrons during the electrode reaction, *F* is the Faraday's constant and θ_Ag_ the coverage of the Ag coverage onto the catalyst surface induced by the backspillover of Ag^+^ ions from the electrolyte.

When the promoting species can take part in the reaction (e.g., O^2−^ in an oxidation reaction or H^+^ in a hydrogenation reaction), the Faradaic efficiency reveals if the process is (sub)Faradaic (i.e., Λ ≤ 1) or if the catalytic reaction exhibits NEMCA behavior (i.e., |Λ| > 1). However, in the case of promoting species that cannot participate in the reaction (e.g., K^+^, Na^+^, or Ag^+^), the Faradaic efficiency has no significant meaning and the promotional index is used. Upon applying a positive (negative) current, the Ag^+^ cations are removed (supplied) from (to) the Pt catalyst surface and, in this way, the θ_Ag_ is decreased (increased).

## Results and discussion

### Electrochemical catalyst characterization

**Figure 2:**
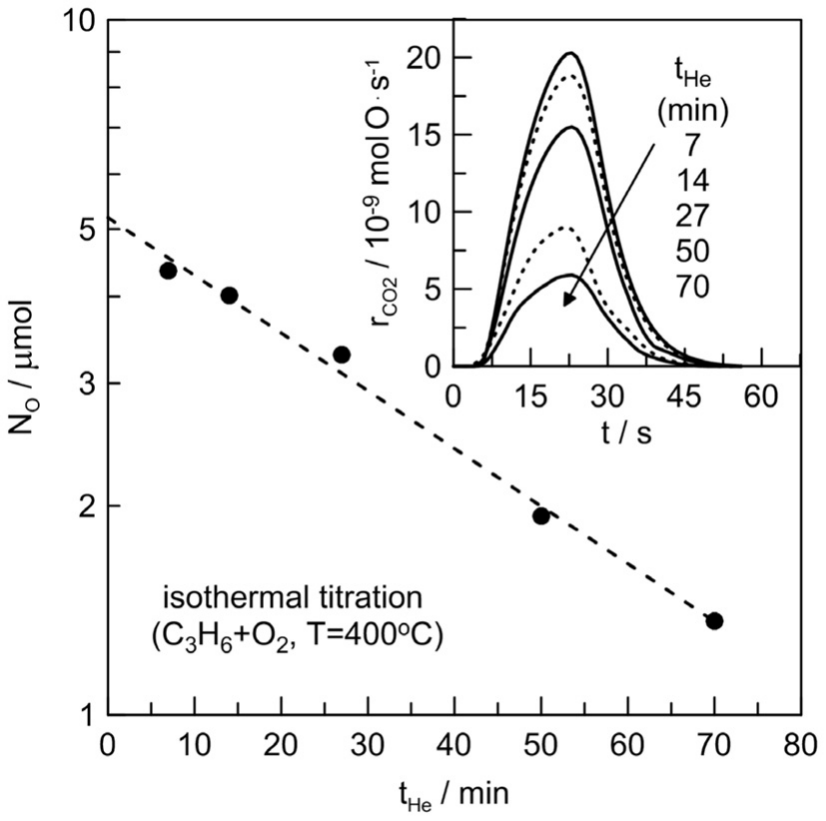
**Isothermal surface titration of oxygen by C_3_H_6_ at 400°C**. Effect of oxygen desorption time, *t*_He_, on the mass of reactive oxygen adsorbed on the Pt/β″-Al_2_O_3_ (Ag^+^) electrochemical catalyst. CO_2_ peaks for each *t*_He_ are also depicted in the index.

The dispersion of the Pt film after catalysis was determined by measuring its reactive oxygen uptake at 400°C via the isothermal titration technique (Vayenas et al., 2001). The electrochemical catalyst was first exposed to O_2_ for 1 h, then purged with pure He for several times, *t*_He_, longer (>10 times) than the reactor residence time (~0.5 min) to remove gaseous O_2_. Subsequently, the reactor was exposed to C_3_H_6_, and the amount of oxygen remaining on the Pt surface (N_*O*_) was obtained by integrating the area of the CO_2_ peak in the reactor effluent (Figure 2). The same procedure was repeated several times by varying the value of *t*_He_. In this way, one can study the kinetics of oxygen desorption and obtains the reactive oxygen catalyst uptake, N_G_, by extrapolating N_*O*_ at *t*_He_ = 0. Figure 2 shows the results of the surface titration measurements at 400°C. The reactive oxygen uptake was *N*_G_ = 5.2 μ mol O. If we attributed this oxygen adsorption only to Pt, this led to a metal dispersion of 50%. This value of dispersion is around two orders of magnitude higher than the values usually observed in electrochemical catalysts (dispersion <0.5%) prepared with similar way (i.e., organometallic paste application annealed at high temperature) (Vayenas et al., 2001). This may indicate that a part of the adsorbed oxygen must be attributed to the presence of silver species in the catalyst/electrolyte interface, and that O-Ag-Pt complexes probably exist. Moreover the dissolution of oxygen on Ag, because of the finite solubility of oxygen in Ag, maybe plays a key role in this system.

The slope in the isothermal titration experiment is also peculiar if we consider that oxygen is only adsorbed on a Pt free of Ag catalyst surface. Indeed, even after 70 min purging with Helium (i.e. *t*_He_ = 70 min) significant amount of oxygen remains onto the catalyst surface (index of Figure 2). For instance, the titration of a Pt/Al_2_O_3_(K^+^) electrochemical catalyst, (sintered at the same temperature than the electrochemical catalyst of this work) performed at 350°C using C_3_H_6_ (de Lucas-Consuegra et al., 2007) gives a the desorption constant, *k*_*d*_, of 5.7·10^−8^ molO/s. In the electrochemical catalyst of the present study, at 400°C, the desorption constant is 50 times smaller (i.e., *k*_*d*_ = 0.11·10^−8^ molO/s). This provides another insight that silver species play an important role in this electrochemical catalyst.

### Catalytic activity measurements

#### Galvanostatic transient experiment

**Figure 3:**
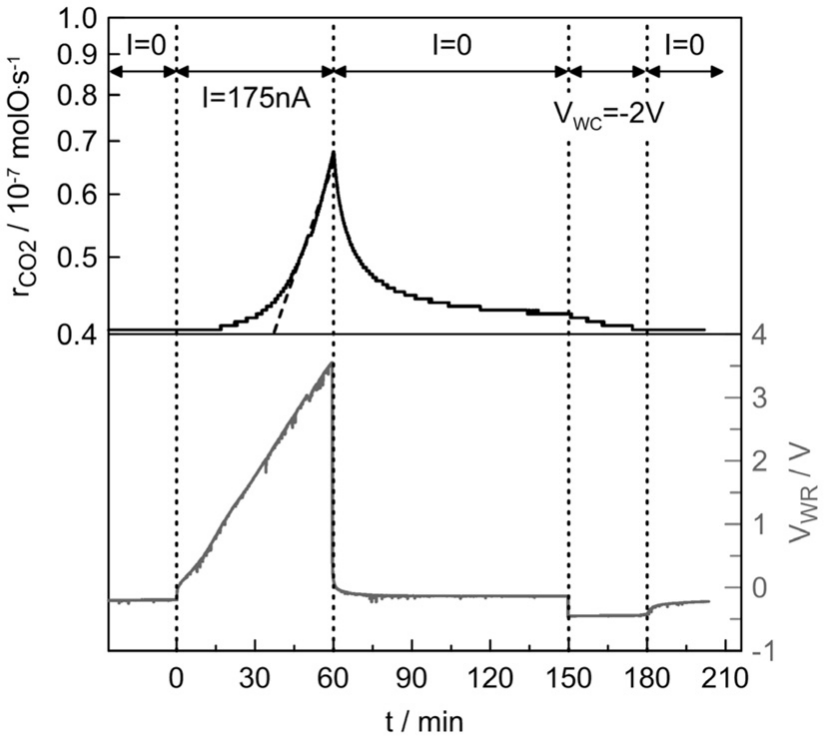
**Catalytic rate and potential responses to step changes of a small (+175 nA) current application and a negative (−2V) potential imposition during C_3_H_8_ oxidation on Pt/β″-Al_2_O_3_ (Ag^+^) at 375°C**. Reactive mixture: C_3_H_8_/O_2_: 2400 ppm/1.2%. Overall flow: 12 L/h.

Figure 3 shows the catalytic rate and potential responses to step changes of a small (+175 nA) current application as well as a negative (−2V) potential imposition during C_3_H_8_ oxidation on Pt/β″-Al_2_O_3_ (Ag^+^) at 375°C. Initially (*t* < 0), the electrical circuit is open and the open-circuit (unpromoted) catalytic rate is 4.0 · 10^−8^ molO/s. At *t* = 0, a small positive current (*I* = +175 nA) is applied between the working and counter electrodes and thus Ag^+^ are removed from the catalyst surface, at the rate *I*/F = 1.8 · 10^−12^ mol/s. This causes (after 1 h) a nearly 70% enhancement in the catalytic rate (ρ = 1.7). The rate increase, Δ*r*, is 31,200 times larger than the rate of Ag^+^ removal from the catalyst surface, thus the apparent Faradaic efficiency Λ equals 31,200, while the promotional index is equal to 1160, however, this value is calculated with the *N*_*G*_ value extracted by the surface titration. Upon current interruption (*t* = 60 min), the catalytic rate decays to a slightly higher value (4.2 · 10^−8^ molO/s) than its initial value over a relaxation time period of several minutes (90 min).

The catalyst potential, UWR, rapidly decays to a value of −145 mV, which is higher than the initial (before polarization) one of −215 mV. When the current application is interrupted, the Ag^+^ ions cannot totally return to the catalyst surface and this causes this difference in the open-circuit values of the catalytic rate and potential between before and after polarization. This behavior has been also observed in EPOC studies with Na^+^ and K^+^ conductors (de Lucas-Consuegra et al., 2007; Kotsionopoulos and Bebelis, 2007), where it was observed that potentiostatic imposition for several minutes can force alkali ions to move and this finally can restore the catalytic rate to its initial value.

Imposition of −2V between the working and the counter electrodes for 30 min was enough for restoring the initial state catalytic rate and after potential interruption the catalyst potential also returned to its initial value. In general the initial state depends on the initial Ag^+^ coverage and thus in the previous catalyst history, however, in this case the system after negative polarization returns to the same state.

Propane oxidation on Pt/β″-Al_2_O_3_ (Ag^+^) exhibits electrophobic behavior (i.e., the catalytic rate increase upon positive overpotential) as reported with Pt/β″-Al_2_O_3_ (Na^+^) and Pt/YSZ(O^2−^) electrochemical catalysts at stoichiometric conditions (Vernoux et al., 2002; Bultel et al., 2004; Kokkofitis et al., 2005, 2007; Kotsionopoulos and Bebelis, 2007; Kambolis et al., 2012). As reported in the literature (Vernoux et al., 2002; Bultel et al., 2004; Kokkofitis et al., 2005, 2007; Kotsionopoulos and Bebelis, 2007) propane and oxygen adsorb competitively on Pt catalyst surface and, as a result, a Langmuir–Hinshelwood type kinetic behavior is observed. Competitive adsorption of oxygen and propane strongly favors oxygen (Vernoux et al., 2002; Bultel et al., 2004; Kotsionopoulos and Bebelis, 2007). This explains positive reaction orders with respect to propane and zero or negative reaction orders with respect to oxygen, reported in the literature (Vernoux et al., 2002; Bultel et al., 2004; Kokkofitis et al., 2005, 2007; Kotsionopoulos and Bebelis, 2007). Therefore, propane deep oxidation on Pt is limited by the propane adsorption. Anodic polarization weakens the Pt-O bonds and therefore increases the propane coverage on the catalyst surface, which simultaneously improves the reaction kinetics.

#### EPOC equation for rate dependence on catalyst potential

A noteworthy aspect of the galvanostatic rate transient, depicted in Figure 3, is that, during the current application (*t* = 0–60 min), the catalyst potential increases almost linearly with the time (except from the initial abrupt increase of 150 mV due to the ohmic losses). This trend is due to extremely low current (near to equilibrium) and absence of parasitic effects of electrode reactions. The catalytic rate starts to increase when the catalyst potential exceeds 1 V (*t* = 15 min) and above 2 V (*t* = 45 min) this increase is exponential with time (the *r*_CO2_ scale is logarithmic).

This behavior (rate exponential increase with time) is, for the first time, observed in galvanostatic EPOC experiments although this in excellent agreement with EPOC theory. EPOC literature (Ladas et al., 1991; Vayenas et al., 2001; Tsampas et al., 2009) has shown that, quite often, over relatively wide (e.g., 0.3–0.5 V) ranges of potential, the catalytic rates depend on catalyst-electrode potential in an exponential manner, similar to the high field approximation of the Butler-Volmer equation, i.e., (Bockris et al., 2000):

(5) $$r/r_{o}=\exp\left(\alpha_{N}\frac{e\Delta U_{WR}}{k_{b}T}\right)=\exp\left(\alpha_{N}\frac{\Delta \Phi }{k_{b}T}\right)$$

where *r*_*o*_ is the unpromoted (i.e., open-circuit) catalytic rate, *k*_*b*_ is the Boltzmann's constant, Δ*U*_*WR*_ is the applied overpotential, ΔΦ is the overpotential-induced change in the catalyst electrode work function and α_*N*_ (typically |α_*N*_| ≈ 0.2–1) is a parameter which is positive for electrophobic reactions (∂*r*/∂Φ > 0). The second equality (5) holds because as shown both theoretically and experimentally [by means of Kelvin probe and UPS work function measurements (Vayenas et al., 1990; Tsiplakides and Vayenas, 2001)], the equality:

(1) $$e\Delta U_{WR}=\Delta \Phi$$

is valid over wide (e.g., 0.5–1 V) catalyst-electrode potential ranges in solid state electrochemistry.

The catalyst potential of the system examined in the present work, linearly increases with time. Therefore, if one considers that the Equation (1) is valid over a potential range, then the work function will also linearly increase with the time and according to Equation (5) the catalytic rate will increase exponentially with the time (as it is experimentally observed).

The slopes of the catalyst potential and rate (in logarithmic scale) of Figure 3 are Δ*U*_*WR*_/Δ*t* = 1 mV/s and Δ1*nr*/Δ*t* = 3.7 · 10^−4^ s^−1^. They are constant for a wide range of time. Therefore, the slope Δ1*nr*/Δ*U*_*WR*_ is equal to 3.7 · 10^−4^ mV^−1^. Combining Equation (5) and this value, one can find that α_*N*_ = 0.82.

The dipole moment of Ag on Pt can be extracted from the data depicted in Figure 3 according to the following procedure. From Faraday's law, it is known that during a galvanostatic experiment the rate of Ag coverage on the catalyst surface is given by Equation (6).

(6) $$\frac{\Delta \theta_{\text{Ag}}}{\Delta t}=\frac{-I/F}{N_{G}}$$

In general the variation in the work function, Φ, with the coverage, θ*j*, of an adsorbate is described by the Helmholtz equation:

(7) $$\Delta \Phi =\frac{eN_{M}}{\varepsilon_{o}}P_{j}\Delta \theta_{j}$$

where *e* = 1.6·10^−19^ C, ε_*o*_ = 8.85·10^−12^ C^2^/J·m, *N*_*M*_ is the surface atom density (e.g., for Pt is 1.53 · 10^19^ atom/m^2^) of the surface under consideration and *P*_*j*_ (C·m) is the dipole moment of adsorbate *j* in the adsorbed state. In a differential form Equation (7) can be written as following:

(8) $$\frac{\Delta \Phi }{\Delta t}=\frac{eN_{M}}{\varepsilon_{o}}P_{\text{Ag}}\frac{\Delta \theta_{\text{Ag}}}{\Delta t}\overset{\Delta \Phi \;=\;e\Delta U_{WR}}{\to }\frac{\Delta U_{WR}}{\Delta t}=\frac{N_{M}}{\varepsilon_{o}}P_{\text{Ag}}\frac{\Delta \theta_{\text{Ag}}}{\Delta t}$$

By combining Equations (6) and (8), one can find the following expression for the dipole moment (assuming that it is constant):

(9) $$\left|P_{\text{Ag}}\right|=\varepsilon_{o}\frac{N_{G}}{N_{M}}\frac{F}{I}\frac{\Delta U_{WR}}{\Delta t}$$

(10) $$\left|P_{\text{Ag}}\right|=94\;\cdot \;N_{G}\;D\;\cdot \;\mu {\text{mol}}^{-1}$$

As it is already mentioned, the *N*_*G*_ value measured with isothermal titration is overestimated by a factor of 100. Therefore, using this value in Equation (10) gives an overestimated value for the dipole moment of Ag on Pt, closed to 490 *D*. But, if we assume dispersion of the order of 0.5%, in relative agreement with literature data (Vayenas et al., 2001), the previous value becomes 4.9 *D* and also the promotional index is equal to 11.6.

#### Potentiostatic transient experiments

**Figure 4:**
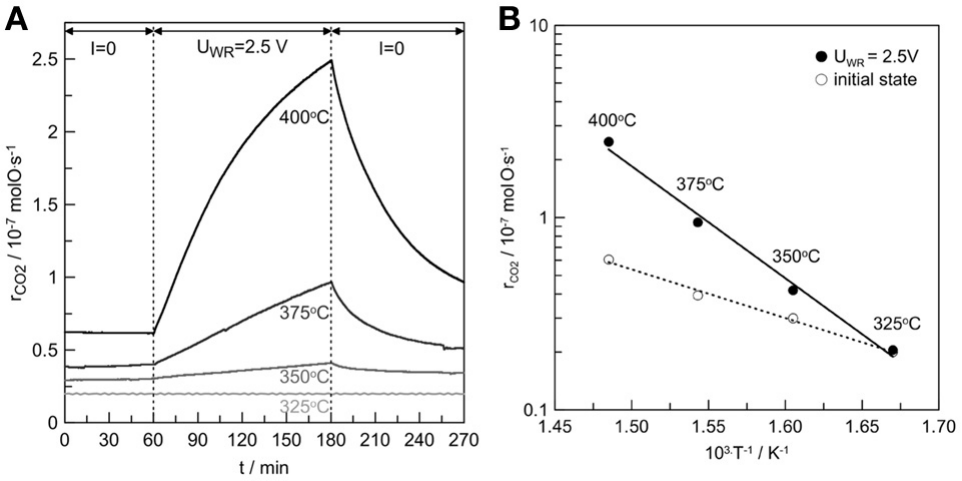
**(A)** Catalytic rate response to a step change of 2.5V during C_3_H_8_ oxidation on Pt/β″-Al_2_O_3_ (Ag^+^) at 325, 350, 375, and 400°C. **(B)** Arrhenius plots at the initial state and upon anodic polarization (*U*_*WR*_ = 2.5 V) conditions. Reactive mixture: C_3_H_8_/O_2_: 2400 ppm/1.2%. Overall flow: 12 L/h.

The catalytic rate response to a step change of a 2.5V is presented in Figure 4A for the temperature range of 325–400°C. Electropromotion becomes more pronounced as the temperature increases. The rate enhancement ratio is equal to 1, 1.3, 1.5, and 4 at 325, 350, 3720, and 400°C, respectively. The above exponential catalytic rate dependence on potential (Equation 5) is usually accompanied by linear variations of the catalytic activation energy, E, with potential, Δ*U*_*WR*_ (Vayenas et al., 2001; Kotsionopoulos and Bebelis, 2007). Figure 4B presents the Arrhenius plots under open circuit and anodic polarization conditions. The apparent activation energy of the reaction was found to be 49 and 110 kJ/mol during initial state and anodic polarization, respectively.

This increase in the apparent activation energy seems to be in contrast with the EPOC concept which should lower the activation energy (Vayenas et al., 2001; Kotsionopoulos and Bebelis, 2007). This strong enhancement of the activation upon positive polarizations coupled with a drastic increase of the catalytic rate could be linked to a change of the nature of the active sites. By increasing the temperature, the positive polarization could reduce silver oxide into active metallic silver sites with a concomitant generation of oxygen ionic species that can migrate onto the catalyst surface. Therefore, significant rate enhancement can be observed. Clearly further investigations should be performed for obtaining better understanding of the processes related with silver oxide decomposition which could generate promoting ionic oxygen species. However, this study is just the first step of the development of a new generation of electrochemical catalysts.

## Conclusions

The development of a new kind of electrochemical catalyst, which permits *in situ* control of Ag^+^ species during propane combustion, was investigated. Encouraging results have shown that significant electropromotion of the catalytic performance can be achieved (rate increase up to a factor of 4). During galvanostatic transient, response of catalytic rate is in excellent agreement with NEMCA theory. Indeed, exponential increase of the rate is observed when the potential linearly increases with time for extremely low currents. The origin of the electrochemical activation upon positive polarizations was tentatively attributed to the electrochemical decomposition of silver oxide which could generate promoting ionic oxygen species.

### Conflict of interest statement

The authors declare that the research was conducted in the absence of any commercial or financial relationships that could be construed as a potential conflict of interest.
